# Supplementary material for: A Temperature-Dependent Viscoplasticity Model for the Hot Work Steel X38CrMoV5-3, Including Thermal and Cyclic Softening under Thermomechanical Fatigue Loading
Source: Materials (Basel). 2023 Jan 21;16(3):994. doi: 10.3390/ma16030994 (PMC9918193; doi:10.3390/ma16030994)
Supplement: Supplementary file 1 [file materials-16-00994-s001.zip › materials-2096653-supplementary.pdf]

materials

Article

A Temperature-Dependent Viscoplasticity Model for the Hot Work Steel X38CrMoV5-3, Including Thermal and Cyclic Softening under Thermomechanical Fatigue Loading

Markus Schlayer [1], Marc Warwas [2], Thomas Seifert [1,*]

[1] Institute for Digital Engineering and Production (IDEeP), Offenburg University of Applied Sciences, Badstraße 24, 77652 Offenburg, Germany
[2] Fraunhofer Institute for Mechanics of Materials IWM, Wöhlerstraße 11, 79108 Freiburg, Germany
* Correspondence: thomas.seifert@hs-offenburg.de

Supplementary Data

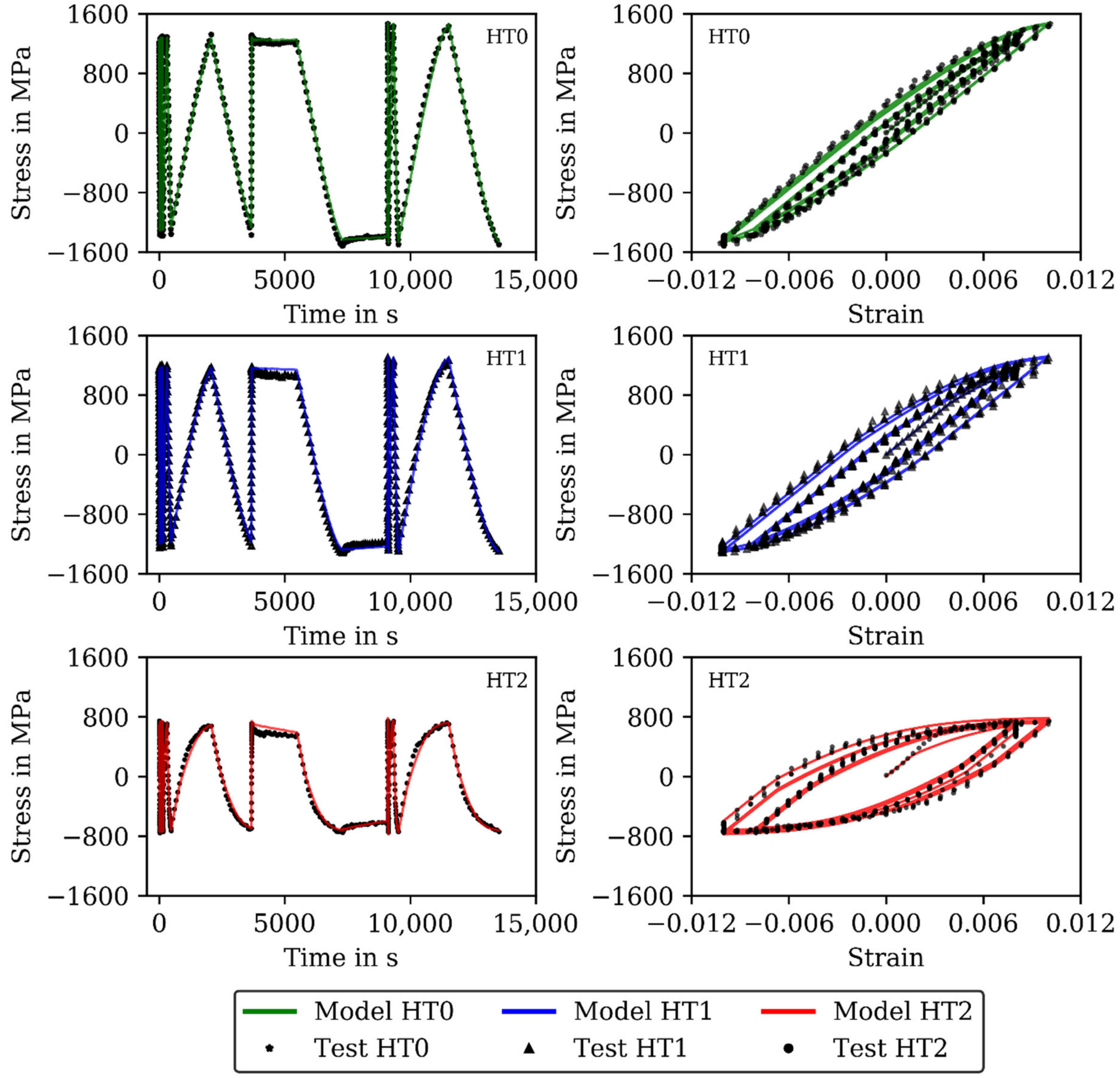

Figure S1. Measured stress and stress calculated with the model of step 1 for the initial (11) cycles of the CLCF tests at 400 °C for the HT0, HT1 and HT2 material. Stress-time diagrams (left) and stress-strain hysteresis loops (right).

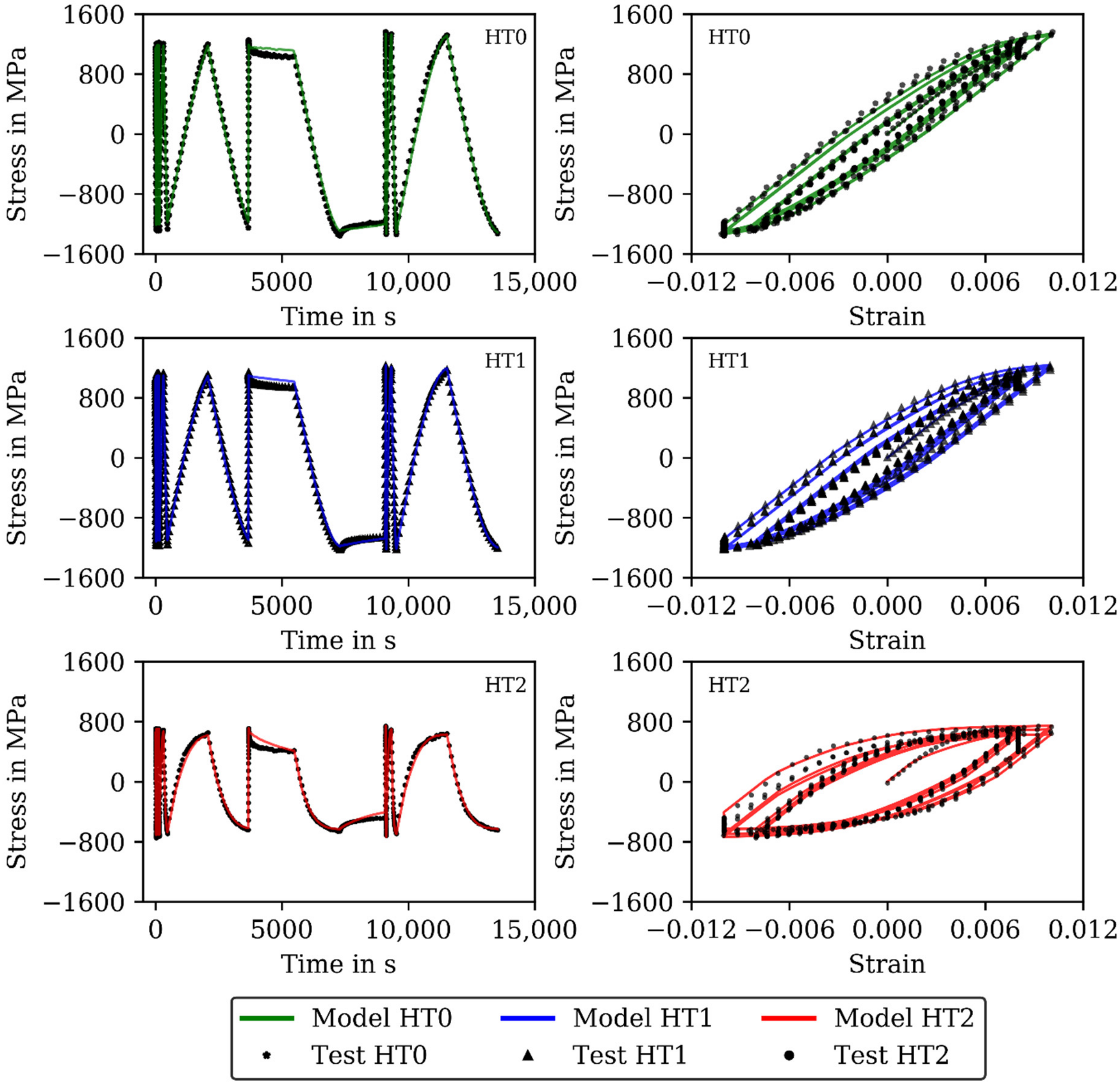

Figure S2. Measured stress and stress calculated with the model of step 1 for the initial (11) cycles of the CLCF tests at 500 °C for the HT0, HT1 and HT2 material. Stress-time diagrams (left) and stress-strain hysteresis loops (right).

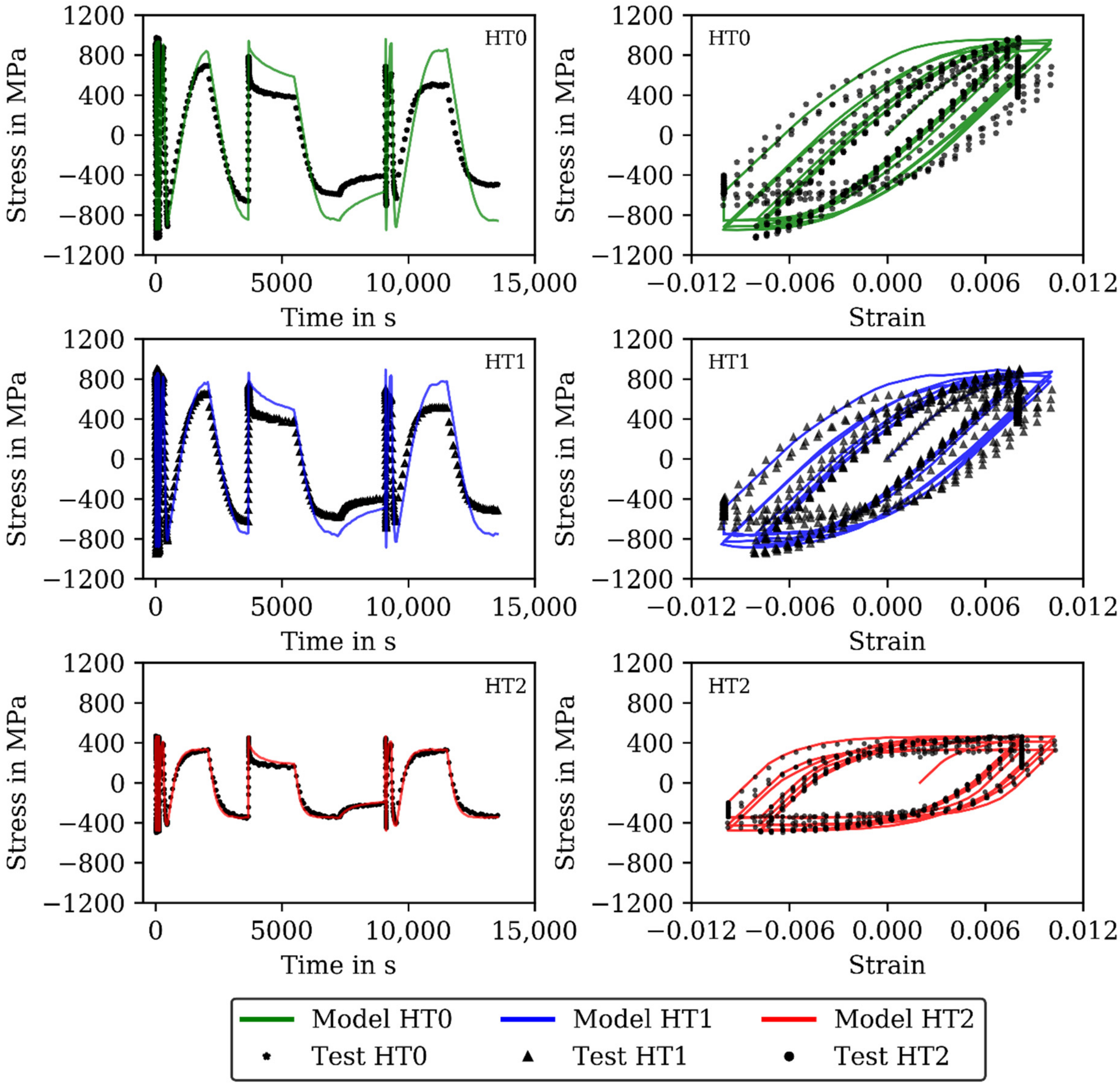

Figure S3. Measured stress and stress calculated with the model of step 1 for the initial (11) cycles of the CLCF tests at 600 °C for the HT0, HT1 and HT2 material. Stress-time diagrams (left) and stress-strain hysteresis loops (right).

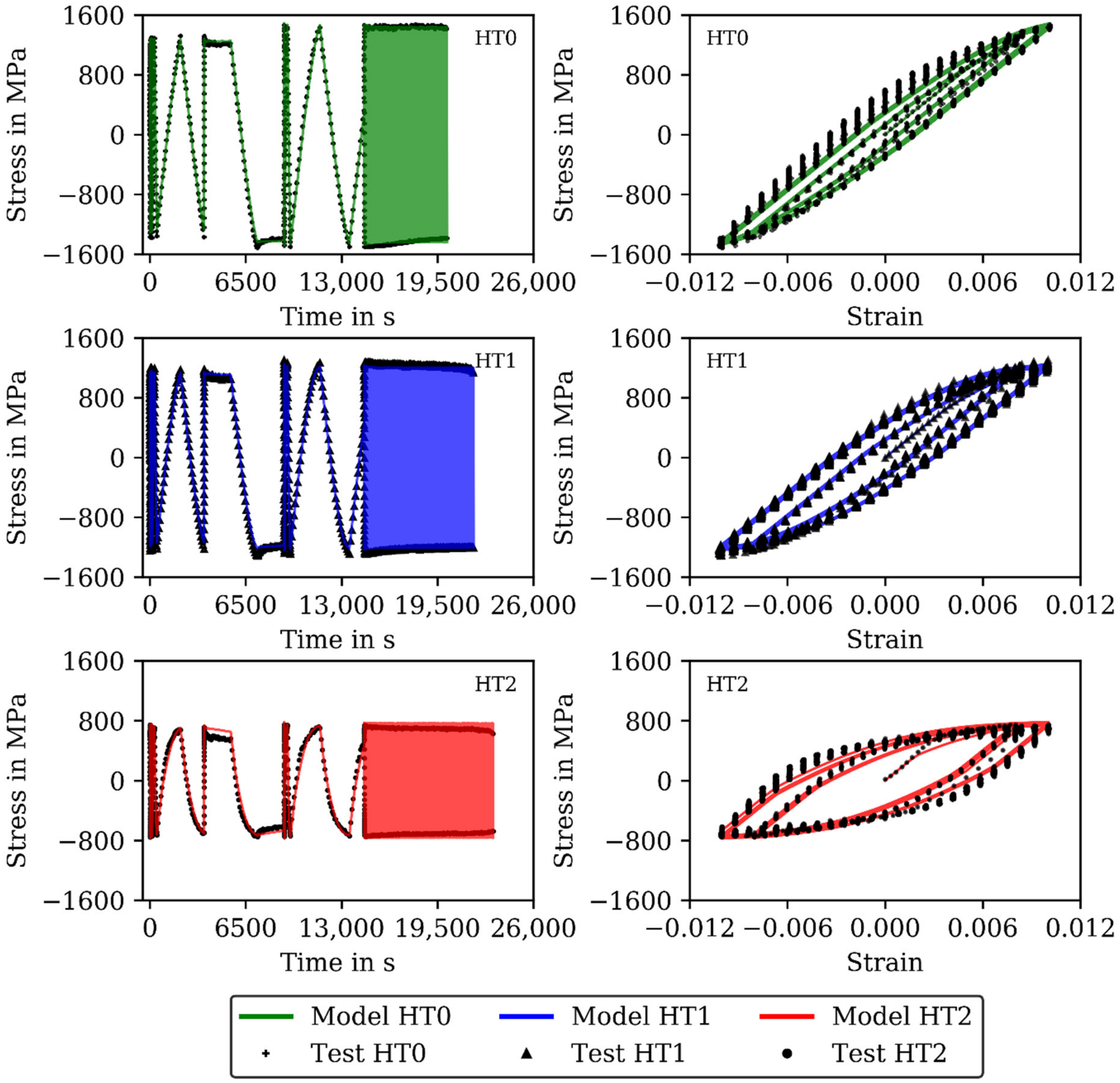

Figure S4. Measured stress and stress calculated with the model of step 2 for all cycles to failure of the CLCF tests at 400 °C for the HT0, HT1 and HT2 material. Stress-time diagrams (left) and stress-strain hysteresis loops (right).

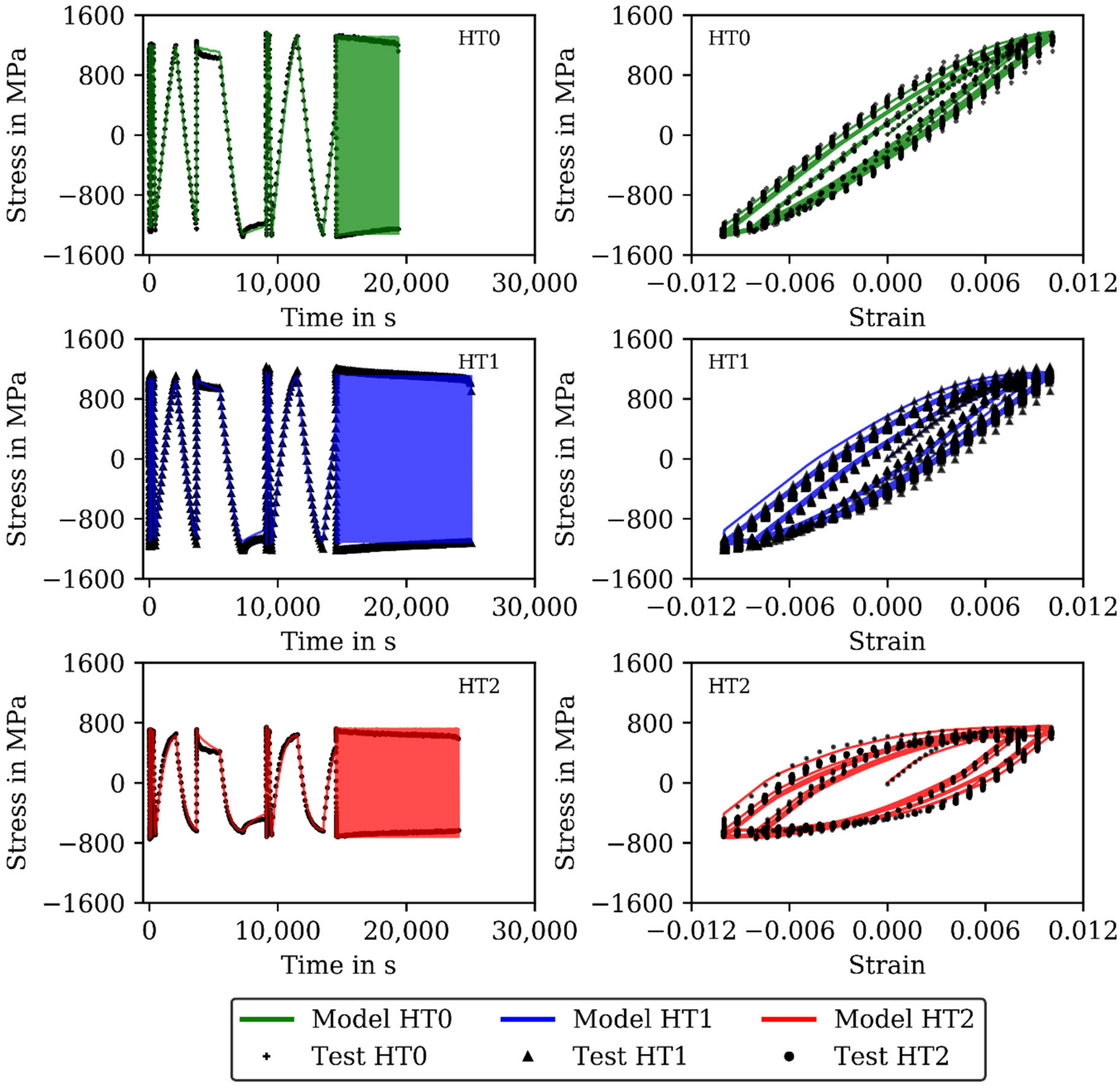

Figure S5. Measured stress and stress calculated with the model of step 2 for all cycles to failure of the CLCF tests at 500 °C for the HT0, HT1 and HT2 material. Stress-time diagrams (left) and stress-strain hysteresis loops (right).

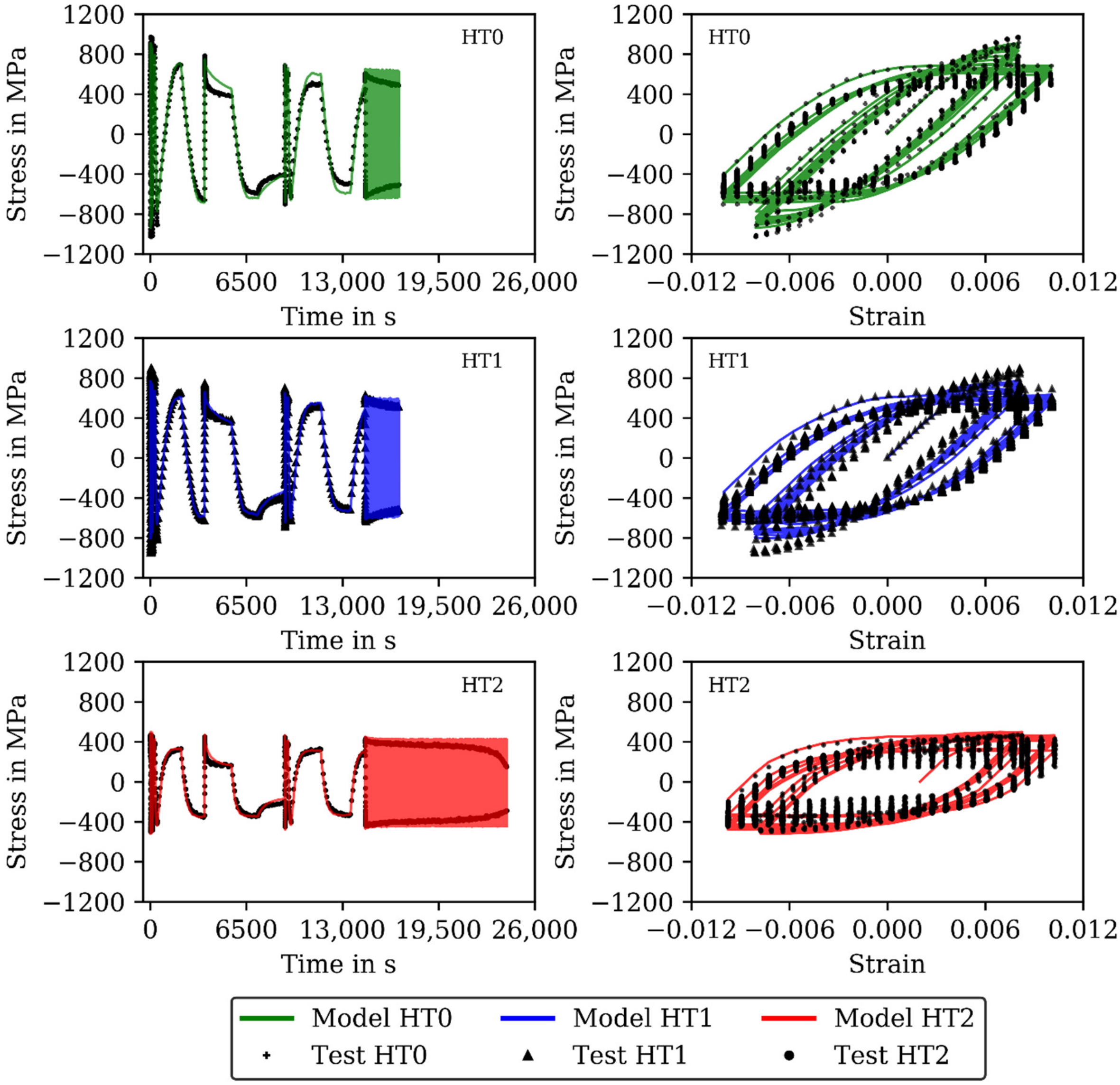

Figure S6. Measured stress and stress calculated with the model of step 2 for all cycles to failure of the CLCF tests at 600 °C for the HT0, HT1 and HT2 material. Stress-time diagrams (left) and stress-strain hysteresis loops (right).

Table S1. Individually determined material properties of step 1 for 20, 400, 500, 600 and 650 °C for the heat treatments HT0, HT1 and HT2 without thermal and cyclic softening.

Temperature in °C	Heat Treatment	E in MPa	R_e in MPa	C in MPa	C_∞ in MPa	K in MPa	n	R
20 °C	HT0	$2.10 \cdot 10^5$	1009	$9.50 \cdot 10^5$	880	10	8	$1.0 \cdot 10^{-5}$
	HT1	$2.10 \cdot 10^5$	790	$6.33 \cdot 10^5$	870	10	7	$2.0 \cdot 10^{-5}$
	HT2	$2.10 \cdot 10^5$	416	$2.00 \cdot 10^5$	520	90	3	$1.0 \cdot 10^{-5}$
400 °C	HT0	$1.75 \cdot 10^5$	771	$8.48 \cdot 10^5$	750	10	8	$5.0 \cdot 10^{-5}$
	HT1	$1.75 \cdot 10^5$	703	$4.70 \cdot 10^5$	650	10	7	$6.0 \cdot 10^{-5}$
	HT2	$1.75 \cdot 10^5$	260	$2.00 \cdot 10^5$	490	436	3	$2.0 \cdot 10^{-4}$
500 °C	HT0	$1.70 \cdot 10^5$	585	$7.48 \cdot 10^5$	750	60	8	$1.0 \cdot 10^{-4}$
	HT1	$1.70 \cdot 10^5$	571	$4.70 \cdot 10^5$	650	104	7	$1.5 \cdot 10^{-4}$
	HT2	$1.70 \cdot 10^5$	230	$2.00 \cdot 10^5$	460	600	3	$7.0 \cdot 10^{-4}$
600 °C	HT0	$1.55 \cdot 10^5$	300	$6.20 \cdot 10^5$	528	300	8	$1.1 \cdot 10^{-3}$
	HT1	$1.55 \cdot 10^5$	263	$4.70 \cdot 10^5$	466	400	7	$1.1 \cdot 10^{-3}$
	HT2	$1.55 \cdot 10^5$	178	$1.30 \cdot 10^5$	203	845	3	$3.0 \cdot 10^{-3}$
650 °C	HT0	$1.47 \cdot 10^5$	120	$3.03 \cdot 10^5$	324	450	8	$2.1 \cdot 10^{-3}$
	HT1	$1.47 \cdot 10^5$	120	$1.30 \cdot 10^5$	190	500	7	$2.1 \cdot 10^{-3}$
	HT2	$1.47 \cdot 10^5$	120	$1.30 \cdot 10^5$	190	1000	3	$4.0 \cdot 10^{-3}$

Table S2. Compiled material properties of step 2 for 20, 400, 500, 600 and 650 °C for the heat treatments HT0, HT1 and HT2 with thermal and cyclic softening.

Temperature in °C	Heat Treatment	R_0 in MPa	R_s in MPa	K_0 in MPa	K_s in MPa	p_0	s_∞
20 °C	HT0	375	635	19	31	0.09	0.20
	HT1	301	477	44	61	0.09	0.20
	HT2	184	228	197	242	0.12	0.20
400 °C	HT0	328	500	19	30	0.08	0.20
	HT1	233	391	26	77	0.08	0.20
	HT2	83	221	67	371	0.10	0.20
500 °C	HT0	236	400	18	31	0.07	0.30
	HT1	167	322	28	100	0.08	0.30
	HT2	60	200	85	527	0.13	0.30
600 °C	HT0	71	287	1	311	0.05	0.60
	HT1	51	240	11	376	0.07	0.50
	HT2	19	165	77	781	0.20	0.35
650 °C	HT0	17	146	4	439	0.20	0.90
	HT1	14	137	12	509	0.21	0.80
	HT2	10	124	65	945	0.25	0.60
